# Antibody Response after Homologous and Heterologous Prime–Boost COVID-19 Vaccination in a Bangladeshi Residential University Cohort

**DOI:** 10.3390/vaccines12050482

**Published:** 2024-04-30

**Authors:** Nihad Adnan, Md. Ahsanul Haq, Salma Akter, S. M. Shafiul Alam Sajal, Md. Fokhrul Islam, Taslin Jahan Mou, Mohd. Raeed Jamiruddin, Fatema Tuz Jubyda, Md. Salequl Islam, Jamsheda Ferdous Tuli, Syeda Moriam Liza, Sharif Hossain, Zinia Islam, Sohel Ahmed, Shahad Saif Khandker, Rubel Hossain, Md. Firoz Ahmed, Mohib Ullah Khondoker, Nafisa Azmuda, Md. Anowar Khasru Parvez

**Affiliations:** 1 Department of Microbiology, Jahangirnagar University, Savar, Dhaka 1342, Bangladesh; salma_akter025@juniv.edu (S.A.); t.j.mou@sms.ed.ac.uk (T.J.M.); fatemajubyda@juniv.edu (F.T.J.); salequl@juniv.edu (M.S.I.); tuli@juniv.edu (J.F.T.); syedamoriamliza@hotmail.com (S.M.L.); firoz@juniv.edu (M.F.A.); azmuda@juniv.edu (N.A.); 2 RNA Biotech Limited, Dhaka 1209, Bangladesh; ahsan@rnabiotech.com.bd; 3 Gonoshasthaya-RNA Biotech Limited, Dhaka 1205, Bangladesh; smshafiulsajal@gmail.com (S.M.S.A.S.); shahadsaifkhandker@gmail.com (S.S.K.); rubelhossain2091997@gmail.com (R.H.); mohibgk@gmail.com (M.U.K.); 4 Institute of Quantitative Biology, Biochemistry and Biotechnology, School of Biological Sciences, University of Edinburgh, Edinburgh EH9 3FF, UK; m.f.islam@sms.ed.ac.uk; 5 Department of Pharmacy, Jahangirnagar University, Savar, Dhaka 1342, Bangladesh; 6 School of Pharmacy, BRAC University, Dhaka 1212, Bangladesh; mohd.raeed@bracu.ac.bd; 7 School of Optometry and Vision Science, Faculty of Medicine and Health, University of New South Wales, Sydney, NSW 2052, Australia; 8 Department of Biotechnology & Genetic Engineering, Jahangirnagar University, Savar, Dhaka 1342, Bangladesh; sharifhossain@juniv.edu (S.H.); ziniaislam@juniv.edu (Z.I.); 9 Department of Biochemistry & Molecular Biology, Jahangirnagar University, Savar, Dhaka 1342, Bangladesh; drsahmed_bmbju@juniv.edu; 10 Gonoshasthaya Samaj Vittik Medical College, Savar, Dhaka 1344, Bangladesh

**Keywords:** heterologous prime and boost, COVID-19 vaccination, humoral immunity, heterologous booster dose

## Abstract

COVID-19 vaccination strategies, including heterologous prime–boost regimens and additional booster doses, aim to optimize immune responses. However, seroepidemiological studies on immune responses to different COVID-19 vaccine types and schedules remain limited. This study investigated antibody levels following homologous and heterologous prime-and-boost COVID-19 vaccination in Bangladesh. In a cohort of 606 participants who received first/second/booster doses of vaccines (AstraZeneca, Moderna, Pfizer-BioNTech, and Sinopharm), anti-spike IgG and anti-nucleocapsid IgG levels were measured. Antibody titer variations with respect to age, gender, intervals between doses, and prior infection status were analyzed. mRNA vaccines elicited the highest antibody levels after homologous and heterologous boosting. The AstraZeneca booster resulted in a sharp titer decline rate of ~0.04 units per day. Second or booster vaccine doses significantly increased antibody levels, especially in males (*p* < 0.05). Older age correlated with higher titers, likely reflecting previous infection, which was further confirmed by the elevation of anti-nucleocapsid IgG levels. About 95.5% of non-Sinopharm recipients were anti-nucleocapsid IgG positive, suggesting prior exposure exceeding self-reported infections (12.5%). mRNA and heterologous COVID-19 boosting enhances humoral immunity over homologous prime–boost vector/inactivated vaccination. However, waning immunity merits further investigation across vaccine platforms.

## 1. Introduction

Coronavirus disease 2019 (COVID-19), declared a pandemic by the World Health Organization (WHO) on 11 March 2020, has become a steady situation after several waves of variants have infected more than 774 million people and claimed more than 7 million lives [1]. In December 2020, the first vaccination program was implemented to resolve the COVID-19 pandemic caused by the SARS-CoV-2 virus [2]. Globally, more than 13.59 billion doses have been delivered as of 21 February 2024 [3]. At least one dose of an authorized vaccination has been administered to 69% of the world’s population, and 32% have been vaccinated with at least one booster dose of a COVID-19 vaccine [3]. As of 26 November 2023, 92% of the total population of Bangladesh had received at least one dose, 86% had completed a primary series, and 42% had received a booster dose [3].

Currently, different types of vaccines, such as DNA, mRNA, non-replicating viral vector, inactivated, live attenuated, subunit, and trained immunity-based vaccines, against SARS-CoV-2 are available [4,5]. So far, Oxford/AstraZeneca, developed by Covishield and Vaxzevria (AZD1222/ChAdOx1 nCoV-19; Andheri, India); Pfizer-BioNTech (BNT162b2, Kalamazoo, MI, USA), Moderna (mRNA-1273, Cambridge, MA, USA); Johnson & Johnson (Ad26.COV2.S, New Brunswick, NJ, USA); Sinopharm (BBIBP-CorV, Beijing, China), Sinovac (CoronaVac, Beijing, China); Sputnik-V (Gamaleya, Moscow, Russia); and Covovax (Novavax, Gaithersburg, MD, USA) have been introduced to the Bangladeshi population [6]. However, initially, four types of vaccines, including COVID-19 mRNA vaccines encoding the S protein of SARS-CoV-2 separately developed by Moderna (mRNA-1273) and Pfizer-BioNTech (BNT162b2); Covishield (AZD1222), a non-replicating viral vector vaccine; and Sinopharm (BBIBP-CorV), an inactivated vaccine, were widely used against SARS-CoV-2 worldwide [5,7,8]. It is commonly understood that vaccines provide protection against infectious agents by eliciting both humoral and cellular immunity, both types of immunity being interrelated. However, in the case of humoral response, vaccine-induced antibodies decline rapidly after the first dosage [9]. Henceforth, except for a few live-attenuated vaccines that protect for extended periods, multiple and booster doses are required for most vaccines to boost levels of antibody responses [10].

Vaccine shortages, particularly in impoverished areas, the advent of novel SARS-CoV-2 variants of concern that are partially resistant to current vaccines, and a number of adverse responses have pushed governments and health authorities towards a heterologous prime-and-boost concept in the administration of COVID-19 vaccines [11]. The heterologous prime-and-boost concept employs heterologous vaccines in prime–booster doses and can potentially mitigate, to an extent, the challenges mentioned above. Post the Delta variant outbreak, some countries accelerated their vaccination programs. To increase the effectiveness and protection of prime vaccine doses, they administered different vaccines as second and booster doses [12]. Bangladesh also incorporated the heterologous prime-and-boost concept into its vaccination program. Although antibody levels after vaccination with AZD1222 (Oxford/AstraZeneca and CoviShield COVID-19 (C19VAZ)) were reported in Bangladesh [13,14], limited data exist after different combinations of heterologous vaccine dosages.

Our study observed the anti-spike-1+ receptor binding domain (S1 + RBD) antibody levels within a cohort following homologous or heterologous prime and booster vaccine administration. We collected samples at random points to investigate the potential association between the number of days between vaccine administration and blood collection, gender, and prior infection with antibody levels.

## 2. Materials and Methods

### 2.1. Ethical Approval

Ethical clearance was obtained from the Biosafety, Biosecurity, and Ethical Committee of Jahangirnagar University [approval number: BBEC, JU/M-2022/COVID-19/2(1)]. A consent form and demographic information, such as age, educational qualification, occupation, and information regarding COVID-19 previous infection and vaccination, were obtained from each participant. No individual was included in the study without consent.

### 2.2. Study Population and Sample Size Estimation

A serosurveillance study conducted by Raqib R et al. in 2021 revealed a weighted seroprevalence of 67.3% for SARS-CoV-2 [15]. Considering this finding with a precision of 0.04% and a 95% confidence interval, the sample size was determined to be 263. A design effect was applied as a correction factor in the sample size calculations to account for potential biases arising from the extensive locality, socioeconomic status (SES), and the unknown transmission rate of SARS-CoV-2. After incorporating a design effect of 2.00, the estimated sample size was increased to 526. The final estimated sample size was adjusted to 585 after factoring in a 10% non-response rate.

In our cross-sectional study, participants were recruited by disseminating invitations across all departments of Jahangirnagar University. Interested individuals were invited to the Department of Microbiology, Jahangirnagar University, from 26 to 30 June 2022. Recruitment of the participants was continued until we achieved the estimated power of 585. However, due to a surge in participant interest at the last moment, we enrolled 608 participants in this study. Willing participants underwent a comprehensive consent process, upon completion of which relevant information and samples were collected from the participants. The information was collected through an online questionnaire with a group of designated study volunteers to streamline the process of participant data input during data collection. After data collection, participants were directed to the sampling booth, where their body temperature, blood pressure, weight, height, and pulse rate were measured, followed by the obtainment of 3 mL blood samples which were stored at −80 °C until testing. A blood sample was collected once from each participant throughout the study period. The study followed the ROSES-1 Statement for influenza (Supplementary Table S1) [16].

### 2.3. Antibody Titer Measurement Assays

In this study, two in-house enzyme-linked immunosorbent assay (ELISA) systems were used to assess the participants’ anti-spike IgG (anti-S1 + RBD IgG) and anti-N IgG antibody levels [17,18,19]. Briefly, ELISA plates were pre-coated with SARS-CoV-2 Spike 1 and RBD proteins (Sino biological, Beijing, China) at a ratio of 6:4, and SARS-CoV-2 nucleocapsid protein (Sino biological, Beijing, China) for anti-spike IgG (anti-S1 + RBD IgG) and anti-N IgG detection. After the serum separation, the serum was diluted (1:100) with dilution buffer and dispensed into wells with positive, negative, and plate controls. After incubation for 15 min and a washing step, horseradish peroxidase-conjugated anti-human IgG (The NativeAntigens, London, UK) at a 1:4000 dilution was added to the wells. After a short incubation and wash, the substrate, 3,3′,5,5′-Tetramethylbenzidine (TMB), was added to each well, followed by a stop solution. The optical density of the final reaction was measured at 450 nm. The antibody level was finally determined by analyzing the OD/cut-off ratio.

### 2.4. Statistical Analysis

The time duration between the blood collection and the last vaccination date was calculated. Frequency tables illustrated vaccination status, demographic features, and COVID-19-related data. A multivariate regression model was used to investigate the variations in antibody titers across different types of administered vaccines and to estimate the mean anti-S1 + RBD-IgG titers. Using the aforementioned regression model, we also evaluated the antibody titers for homologous and heterologous prime and booster vaccinations, the association of antibody titers, and the number of days between receipt of the last vaccine, receipt of the booster dose, and receipt of the second booster dose. All the regression models were adjusted by potential covariates, such as age, gender, occupation, anti-N IgG results (categorical, positive, and negative), duration since the last vaccine (in days), BMI, and blood pressure. A linear regression model was used to observe the association between the time since the last vaccine and anti-S1-RBD IgG and anti-N IgG levels. Statistical analyses were carried out using Stata 15 (Stata Corp, LP, College Station, TX, USA), and graphical presentations were generated using GraphPad Prism (version 8.3.2).

## 3. Results

### 3.1. Demographics

A total of 608 participants, including both males [n = 387 (63.7%)] and females [n = 221 (36.4%)], participated in the study. The participants were of different ages (18 to 65 years) with various educational backgrounds and occupations. Participants’ body mass index (BMI), comorbidities, pulse rate, and COVID-19 infection data were collected and recorded (Table 1). In our study, approximately three-fourths of the population were within the normal range, while one-fourth were within the overweight range. Additionally, 60% of our study population comprised university students or graduates. When observed for comorbidities within each gender population, 32% were male and 24.4% were female, such that the frequency of comorbidities among genders was similar.

Fever and pain were the main self-reported adverse events among patients. In percentage terms, Moderna (61.1%) scored highest for adverse events, while Pfizer-BioNTech (10.5%) scored lowest. The lower number of reports of adverse events for Pfizer-BioNTech can be attributed to the low number of participants (n = 19). Sinopharm (n = 269) had the lowest number of reports (13.4%) of fever and pain.

### 3.2. Vaccine Groups

Among the 608 participants, two did not receive any vaccine and thus were excluded from the study for further analysis. The study participants received varying doses of vaccines: 17 (2.81%) received only a first dose, while 227 (37.3%) and 362 (59.5%) received second and booster doses, respectively (Table 2). The mean numbers of days between the first and second and between the second and booster vaccines were 57.3 ± 35.1 and 102.2 ± 42.9 days, respectively. The majority of the participants acquired the following vaccines or a combination thereof: AstraZeneca (vector), Moderna (mRNA), Pfizer-BioNTech (mRNA), and Sinopharm (inactivated). Most of the participants received AstraZeneca as first [n = 282 (46.5%)], second [n = 280 (46.1%)], and booster doses [n = 208 (57.5%)]. Except for booster doses, following AstraZeneca, Sinopharm was the second most received vaccine among the participants as first [n = 269 (44.2%)] and second doses [n = 255 (41.9%)], followed by Moderna and Pfizer-BioNTech. As for booster doses, after AstraZeneca, Moderna [n = 86 (24.0%)] was most frequently received, followed by Pfizer-BioNTech [n = 57 (15.9%)] and Sinopharm [n = 4 (1.12%)]. Only three (0.84%) participants received a vaccine other than these four major types for the booster dose (Table 2).

### 3.3. Anti-Nucleocapsid Antibody Assessment

Upon determination of anti-N IgG levels, it was found that 571 (93.9%) participants were anti-N IgG positive and that 22 (3.6%) were negative. After elimination of the Sinopharm-vaccine recipients, among the remaining 262 participants, 257 (95.5%) and 5 (1.9%) were identified as anti-N IgG positive and negative, respectively.

### 3.4. Anti-Spike IgG (Anti-S1 + RBD IgG) Antibody Levels Post-Second Dose

**Figure 1:**
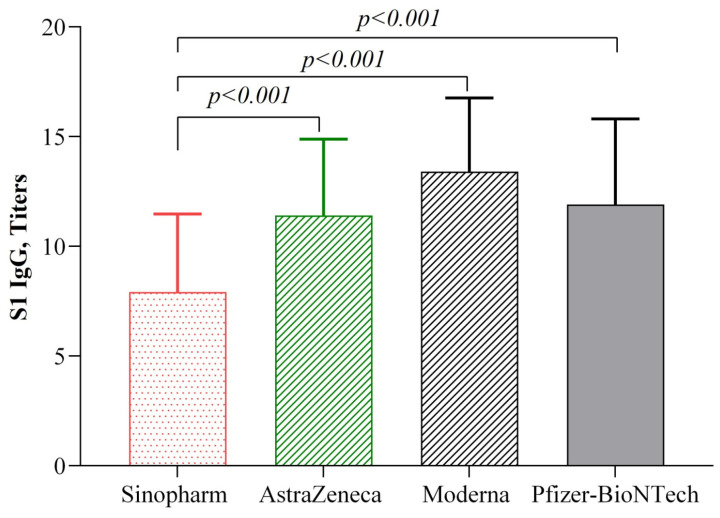
Antibody titers among the vaccinated participants in different vaccine groups after receiving a second vaccine dose. The multivariate regression model was used to estimate the *p*-values, and the regression model was adjusted by age, gender, occupation, duration since receiving the last vaccine (in days), BMI, and blood pressure.

Following the administration of the same vaccine as first and second doses, individuals vaccinated with the Moderna vaccine exhibited notably elevated levels of anti-S1 + RBD IgG (13.4 ± 3.36) which surpassed those of the participants who received AstraZeneca (11.4 ± 3.48) and Sinopharm (7.92 ± 3.55) vaccines (Figure 1). Conversely, recipients of the Pfizer-BioNTech vaccine (11.9 ± 3.91) demonstrated significantly higher levels of anti-S1 + RBD IgG only when compared to those who received the Sinopharm vaccine (*p* < 0.001).

### 3.5. Anti-Spike IgG (Anti-S1 + RBD IgG) Antibody Levels Post-COVID-19 Vaccine Homologous and Heterologous Prime and Booster Doses

**Figure 2:**
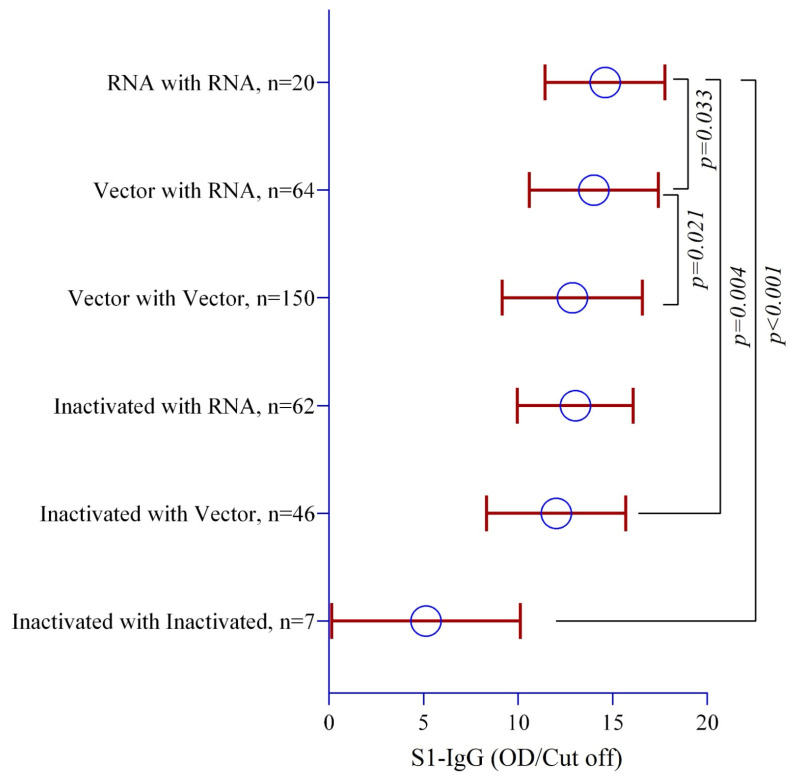
The differences in antibody titers after receiving homologous and heterologous prime–booster doses. The multivariate regression model was used to estimate the *p*-values, and the regression model was adjusted by age, gender, occupation, duration since receiving the last vaccine (in days), BMI, and blood pressure.

A comparison of homologous vaccinations, where individuals received the same vaccine for their first, second, and booster doses, revealed significantly elevated antibody concentrations among mRNA vaccine recipients compared to those who received vector-based (*p* = 0.021) and inactivated vaccines (*p* < 0.001) (Figure 2). Heterologous vaccination denotes the administration of the same vaccine for the first and second doses, followed by a different vaccine for the booster dose. Our findings indicate that mRNA vaccines elicited the highest antibody titers when administered as heterologous booster doses compared to vector-based and inactivated vaccines (Figure 2).

### 3.6. COVID-19 Vaccine-Wise Waning of Anti-Spike IgG (Anti-S1 + RBD IgG) Antibody Levels

**Figure 3:**
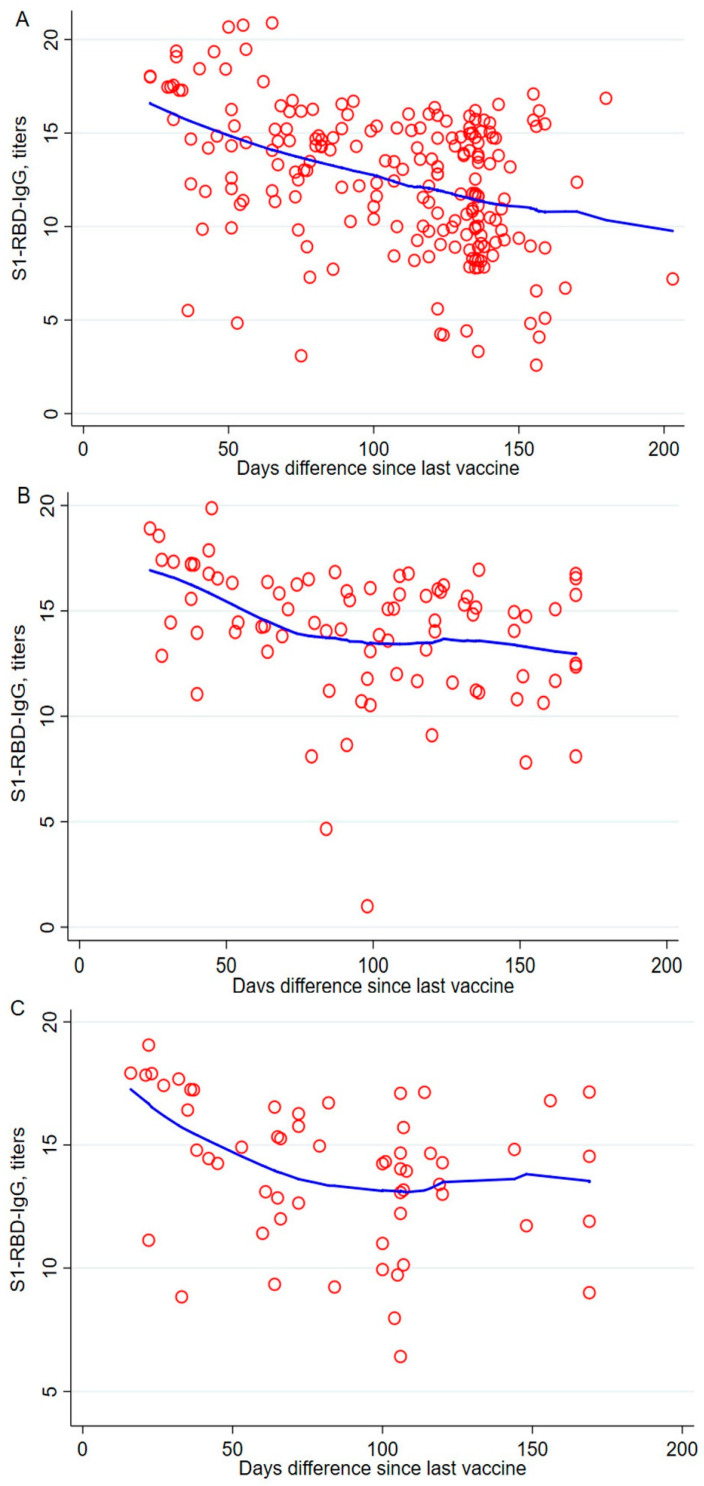
Association of antibody titers and number of days since receiving the last vaccine after a booster dose of the vaccine. A multivariate regression model was used to estimate the *p*-values. The regression model was adjusted by age, gender, education, occupation, and number of days since the last vaccine was received. (**A**) AstraZeneca-vaccinated individuals. (**B**) Moderna-vaccinated individuals. (**C**) Pfizer-BioNTech-vaccinated individuals.

To assess different vaccine-wise changes up to 220 days after receiving a booster dose, we stratified the analysis with the participants’ respective vaccine booster doses. We excluded Sinopharm from this analysis, as only a few participants received it as a booster dose. A sharp decline was noted in the AstraZeneca vaccine recipients, and each day that passed since receiving the vaccine was associated with a 0.04 titer decline (95% CI = −0.05, −0.03, *p* < 0.001) (Figure 3A). The Moderna- and Pfizer-BioNTech-vaccinated participants also showed a significant decline of 0.02 and 0.03 titers, respectively (Figure 3B,C).

### 3.7. Association of Gender with Anti-Spike IgG (Anti-S1 + RBD IgG) Antibody Levels

We assessed the vaccine dose-specific increase in anti-S1 + RBD IgG levels among participants who received second and booster doses compared to those who received only a first dose. Across all participants, it was noted that, following the second dose, there was a significant increase of 2.47 units (95% CI = 0.59, 4.34, *p* = 0.010), and after the booster dose, a substantial increase of 6.83 units (95% CI = 4.97, 8.68, *p* ≤ 0.001) in anti-S1 + RBD IgG levels compared to those who received an initial dose only (Table 3). This elevation in anti-S1 + RBD IgG levels after receiving second and booster doses, in contrast to a first dose only, remained statistically significant for male participants. However, no significant association was observed for female participants (Table 3).

The multivariate regression model was used to estimate the *p*-values, and the regression model was adjusted by age, gender, occupation, and duration since receiving the last vaccine (in days).

### 3.8. Effect of COVID-19 Vaccination on the Persistence of Anti-Spike IgG (Anti-S1 + RBD IgG) Antibody Levels

**Figure 4:**
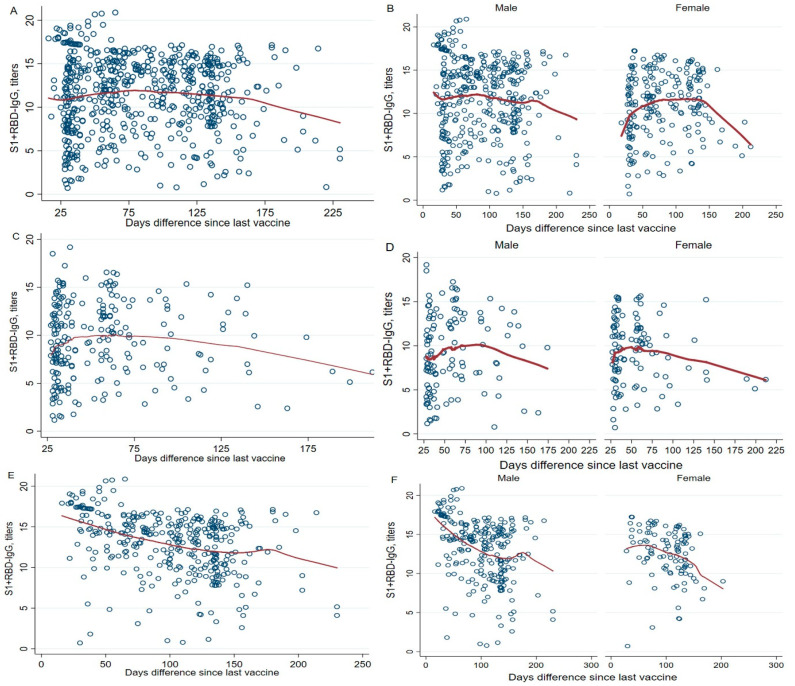
Association of antibody titers with the number of days since receiving the last vaccine after second and booster doses of the vaccine, stratified by gender. A multivariate regression model was used to estimate the *p*-values, and the regression model was adjusted by age, gender, education, occupation, and days since the last vaccine was received. Anti-N IgG results (categorical, positive, and negative) (**A**) for all participants, (**C**) after receipt of a second dose of a vaccine, and (**E**) after receipt of a booster dose of a vaccine. (**B**,**D**,**F**) show the gender stratifications for (**A**,**C**,**E**), respectively.

We computed the duration between vaccine delivery and the date of sample collection in days. Upon analyzing the data of all participants, it was observed that each additional day post-vaccination decreased antibody titers by 0.01 units (*p* = 0.045) (Figure 4A). This association was similarly identified in male participants (*p* = 0.006) (Figure 4B). Furthermore, when examining participants who received a second vaccine dose and the duration in days, the significance was evident solely among female participants. Specifically, a one-day increase was associated with a decrease in antibody titers by 0.01 units (*p* = 0.048) (Figure 4D).

For participants who received a booster dose, the decline in antibody titers remained statistically significant for male and female participants. The reductions were 0.03 units (*p* < 0.001), 0.04 units (*p* < 0.001), and 0.02 units (*p* = 0.013), respectively (Figure 4E,F).

### 3.9. Association of Age with Anti-Spike IgG (Anti-S1 + RBD IgG) Antibody Levels

**Figure 5:**
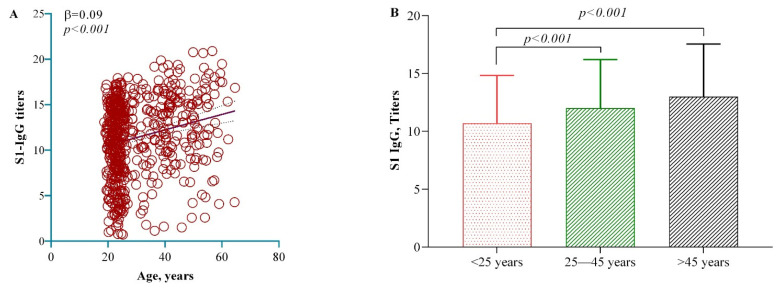
Association of age ((**A**) continuous and (**B**) categorical) with anti-S1 + RBD IgG titers. A linear regression model was used to estimate the *p*-values.

A noteworthy positive association was observed between age and anti-S1 + RBD IgG titers. For each additional year of age, there was a corresponding increase of 0.09 units in anti-S1 + RBD IgG levels (95% CI = 0.06, 0.12, *p* < 0.001) (Figure 5A). This association retained significance even after age stratification; individuals aged 45–65 and within the 25–45 age group exhibited significantly higher antibody titers than those aged 18–25 (Figure 5B).

**Figure 6:**
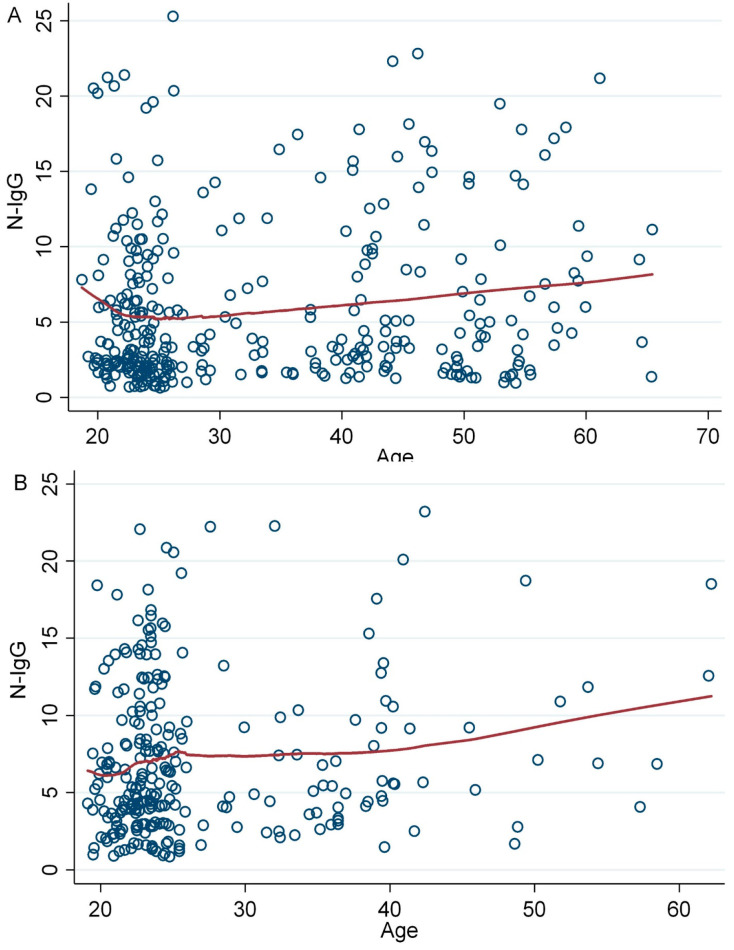
Association between age and anti-N IgG levels among (**A**) mRNA or vector vaccine recipients and (**B**) Sinopharm only vaccine recipients. A simple linear regression model was used to evaluate and check the association.

Interestingly, age was positively associated with anti-N IgG levels among individuals who received either mRNA or vector-based vaccines (β-coff = 0.06, 95% CI= 0.01, 0.10, *p* = 0.011) (Figure 6A). Likewise, recipients of the Sinopharm vaccine exhibited a positive association with anti-N IgG levels (*p* = 0.047) (Figure 6B).

## 4. Discussion

The in-house kit utilized in this study, the anti-Spike IgG (anti-S1 + RBD IgG) kit, was previously clinically evaluated under the Directorate General of Drug Administration (BMRC/NREC/2019-2022/48; date: 17/01/2021) at the International Centre for Diarrhoeal Disease Research, Bangladesh (ICDDR, B) (PR-20080; date: 15 October 2020). The evaluation was carried out using the Elecsys^®^ Anti-SARS-CoV-2 S Immunoassay Kit (Roche Diagnostic GmbH, Mannheim, Germany) as a comparator assay, which exhibited a 92.3% positive agreement rate with a vesicular stomatitis virus (VSV)-based pseudo-neutralization assay. From this trial, the anti-Spike IgG (anti-S1 + RBD IgG) kit showed 98.2% positive agreement with the Elecsys^®^ Anti-SARS-CoV-2 S Immunoassay Kit and a sensitivity of 97.8%. Due to the high positive agreement and the high sensitivity, the kit was implemented in the study [19].

The characteristics of the antibody response induced by a vaccine are mainly dependent on the type of vaccine, besides other factors, such as the gender, health condition, or genetics of recipients [9,20]. Modifying nucleosides, thus increasing translation and down-activating innate immune response, has been a promising improvement in mRNA vaccine technology [21,22]. Moreover, efficient in vivo delivery systems, such as lipid nanoparticle-based mRNA delivery, have given this type of vaccine a major advantage over other vaccine types [23]. On the other hand, recent innovations in genetically engineering adenoviral vectors have led to improved vaccine designs that can overcome limitations faced by early adenoviral-based vaccines. Specifically, molecular modifications of vectors have enabled faster development of vaccines that elicit more potent and durable immune responses [24].

Studies have reported that mRNA vaccines induce stronger humoral immunity than vector-based and conventional vaccines [25,26]. The Moderna and Pfizer-BioNTech COVID-19 vaccines are mRNA vaccines that encode the spike glycoprotein of SARS-CoV-2 [27]. As they both encode the S antigen, after successful vaccination, the mRNA directly translates into S protein, which stimulates the body’s immune system as a foreign particle, ultimately upregulating the production of anti-S antibodies, rather than any other types of antibodies against SARS-CoV-2, which are crucial for virus neutralization [28,29]. AstraZeneca also targets the spike glycoprotein, but it minimizes immunogenicity and reactogenicity compared to the Pfizer-BioNTech and Moderna vaccines [5,29]. On the other hand, the Sinopharm vaccine was designed with an inactivated whole virus, which creates a variety of viral antigen interactions with the immune system after vaccination [30]. Similar to other studies, our study also observed lower humoral immunity with the Sinopharm vaccine compared to mRNA and vector-based vaccines after a homologous second dose (Figure 1).

The “mix-and-match” concept of heterologous vaccine dosages against COVID-19 was initially used due to the shortage of vaccines, lack of transportation and storage facilities, and so on [31]. However, heterologous vaccine dosage was found to boost seroconversion significantly [31,32]. Our data also demonstrated that prime–boost doses of mRNA vaccines achieved the highest anti-S1 + RBD IgG levels compared to vector-based vaccines and inactivated vaccines (Figure 2). Moreover, when mRNA was used as a heterologous booster dose compared to a vector-based vaccine, the former induced higher humoral immunity. Similar observations have been reported, showcasing the effective humoral immunity of future vaccine strategies against infectious agents, like Ebola virus, malaria, human immunodeficiency virus 1, Nipah virus, etc., in prime–booster vaccination programs with mRNA vaccines [33]. Furthermore, around 200 days post-vaccination, we found a sharp waning of anti-S1 + RBD IgG levels with homologous prime–boost AstraZeneca vaccines compared to mRNA vaccines (Figure 3).

Among the 608 study participants, 590 received prime or prime–booster doses of four different types of COVID-19 vaccines (Table 1 and Table 2). Overall significant increases in anti-S1 + RBD-IgG levels of 2.47 units (95% CI = 0.59, 4.34, *p* = 0.010) and 6.83 units (95% CI = 4.97, 8.68, *p* ≤ 0.001) were found in those participants who received second and third doses, respectively, compared to those who received an initial dose only (Table 3). Interestingly, this phenomenon was significant for the male participants, especially for the third dose (*p* < 0.001), but it was found to be insignificant for female participants (*p* = 0.127) (Table 3). Our observation contrasts with other studies, where female participants showed higher antibody titers post-second dose, though it should be noted that the participants in those studies were mainly administered inactivated vaccines [34,35,36]. However, observations for the BNT162b2 vaccine were similar to those in our study, in which there was a significant increase in antibody titers post-second dose in males compared to females aged 21–30 [37].

The rate of decline in antibody titers is a significant observation in cohort studies. Among the vaccines, a 33.89% decline at six months was reported for inactivated vaccines [38]. In the case of adenoviral vector-based vaccines, women exhibited a faster decay in antibody levels of 53% compared to 12% for men [39]. Since the blood was collected after a median post-vaccination period of about three months, we observed lower antibody titers in female participants compared to males, which may suggest a rapid decline in antibody titers in females within populations receiving inactivated or adenoviral vaccines (Figure 4).

Seroprevalence studies for vaccines in an active epidemic or pandemic can provide mixed results, as natural infection and vaccine-induced immunity may cause combined effects. Although only 76 (12.5%) participants among the 608 previously tested RT-PCR positive, we determined that 95.5% were anti-N IgG positive after eliminating the Sinopharm vaccine recipient, indicating that most of the studied population were naturally infected. This may have been due to mild infection where patients completely ignored their physical complications or may have been asymptomatic [40].

Age was positively correlated with antibody titers in this study (Figure 5). However, other groups have reported that antibody titers for COVID-19 vaccines decrease with age [41,42]. When we tested for anti-N IgG titers in the participants, we also found a positive correlation (Figure 6). This explains how, with age, the risk of COVID-19 infection increases and thus N-IgG expression levels increase. As a result, natural infection can mask the exact effect of age on vaccine seroprevalence. Kusunoki et al. also reported a similar observation, where many people in Japan received booster vaccinations after spontaneous infection but did not show an increase in antibody titers [43].

The study has several key limitations that constrain the strength of its conclusions. Firstly, the small sample size of only 608 participants from a specific region limits the robustness of the statistical analysis and the generalizability of the findings. Secondly, most participants had prior COVID-19 infection, which may have influenced immune responses to vaccination, potentially confounding the results. Thirdly, the study only had a limited long-term follow-up of 200 days, which is insufficient to fully understand the waning of immunity over time. Fourthly, the study relied solely on measurements of humoral antibody levels as the metric of immunity, without recourse to data on cellular immune responses or real-world vaccine effectiveness and protection. Fifthly, there was no documentation of the adverse events related to vaccination due to the scope of the study, which assessed the antibody levels in individuals who received vaccinations. Lastly, the differences in responses by vaccine types, especially mRNA vaccines, could not be fully explored, as most participants received inactivated or adenoviral vector vaccines. These limitations suggest that further research is needed to strengthen the conclusions drawn from this study.

## 5. Conclusions

This study from Bangladesh suggests that mRNA and viral vector COVID-19 vaccines may generate stronger antibody responses than inactivated vaccines. It also indicates that heterologous boosting could enhance immunogenicity. Although limited by factors like a small sample size and a short follow-up, this study lays the groundwork for larger, longer-term research to explore the durability of immune responses across various COVID-19 vaccines. With more data, the trends observed here could support strategies to optimize current and future vaccine regimens for better protection. Further investigation is necessary to build on these initial findings and understand the comparative performance of COVID-19 vaccines.

## Figures and Tables

**Table 1:** Demographic characteristics of the study participants.

Variables	Outcomes
Age	30.6 ± 11.3
Age category	
18–25 years	329 (54.1%)
25–45 years	191 (31.4%)
45–65 years	88 (15.5%)
Gender	
Male	387 (63.7%)
Female	221 (36.3%)
Education	
Below HSC	37 (6.1%)
HSC	210 (34.5%)
Bachelor	180 (29.6%)
Masters	106 (17.4%)
PhD	75 (12.3%)
Occupation	
Student	375 (61.7%)
Teacher	122 (20.1%)
University staff	111 (18.3%)
BMI, Kg/m^2^	
Underweight (<18.5)	(0.70%)
Normal (18.5–24.9)	439 (72.2%)
Overweight (25–29.9)	161 (26.5%)
Comorbidities *	
Overall	178 (29.3%)
Male	124 (32.0%)
Female	54 (24.4%)
Fever and pain distribution after the first dose of vaccination	
AstraZeneca	114 (40.4%)
Moderna	22 (61.1%)
Pfizer-BioNTech	2 (10.5%)
Sinopharm	36 (13.4%)
Previously infected with COVID-19	76 (12.5%)

Data are presented as means ± SDs or as numbers with percentages in parentheses. * Comorbidities: hypertension, pulmonary TB, diabetes, and COPD.

**Table 2:** COVID-19 vaccination history.

Doses of Vaccine	Overall (n = 606)	Male (n = 385)	Female (n = 221)
Only received first dose	17 (2.81%)	16 (4.16%)	1 (0.45%)
Only received both first and second dose	227 (37.3%)	118 (30.7%)	109 (49.3%)
Received booster dose	362 (59.5%)	251 (65.2%)	211 (50.2%)
Days between Vaccines			
Days between first and second doses	57.3 ± 35.1	58.2 ± 34.5	56.2 ± 35.8
Days between second and booster doses	102.2 ± 42.9	101.8 ± 45.3	102.7 ± 36.6
Vaccine Types			
Vaccine name (first dose)			
AstreZeneca	282 (46.5%)	191 (49.6%)	91 (41.2%)
Moderna	36 (5.94%)	19 (4.94%)	17 (7.69%)
Pfizer-BioNTech	19 (3.10%)	11 (2.86%)	8 (3.62%)
Sinopharm	269 (44.2%)	164 (42.6%)	105 (47.5%)
Vaccine name (second dose)			
AstreZeneca	280 (46.1%)	189 (51.2%)	91 (41.4%)
Moderna	36 (5.90%)	19 (5.15%)	17 (7.73%)
Pfizer-BioNTech	18 (3.00%)	11 (2.98%)	7 (3.18%)
Sinopharm	255 (41.9%)	150 (40.7%)	105 (47.7%)
Vaccine name (booster dose)			
AstreZeneca	208 (57.5%)	141 (56.2%)	67 (60.4%)
Moderna	86 (14.1%)	59 (23.5%)	27 (24.3%)
Pfizer-BioNTech	60 (9.90%)	45 (17.9%)	15 (13.5%)
Sinoppharm	8 (1.30%)	6 (2.39%)	2 (1.80%)

Data are presented as numbers with percentages in parentheses or as means ± SDs.

**Table 3:** Association of vaccination doses with antibody titers stratified by gender.

Variables	Overall (n = 590)		Male (n = 375)		Female (n = 215)	
	β-Coefficient (95% CI)	*p*-Value	β-Coefficient (95% CI)	*p*-Value	β-Coefficient (95% CI)	*p*-Value
First dose	Ref.		Ref.		Ref.	
Second dose	2.47 (0.59, 4.34)	0.018	2.35 (0.28, 4.43)	0.035	1.24 (−5.37, 7.84)	0.855
Booster dose	6.83 (4.97, 8.68)	<0.001	6.96 (4.92, 9.00)	<0.001	5.19 (−1.49, 11.9)	0.127

## Data Availability

The manuscript includes all data, presented in both tables and figures. Should further data be needed, they can be obtained by contacting the corresponding author.

## Supplementary Materials

The following supporting information can be downloaded at: https://www.mdpi.com/article/10.3390/vaccines12050482/s1, Table S1: ROSES-I statement checklist.
