# Supplementary material for: Antibody Response after Homologous and Heterologous Prime–Boost COVID-19 Vaccination in a Bangladeshi Residential University Cohort
Source: Vaccines (Basel). 2024 Apr 30;12(5):482. doi: 10.3390/vaccines12050482 (PMC11125736; doi:10.3390/vaccines12050482)
Supplement: Supplementary file 1 [file vaccines-12-00482-s001.zip › vaccines-2910900-supplementary.pdf]

Supplementary table: ROSES-I statement checklist

Item	Item Number	ROSES-I items	Checkbox	Comments
Title and abstract	1	ROSES-I 1.1: The term “seroepidemiologic,” “seroepidemiology,” “seroprevalence,” or “seroincidence” should be applied to the study in the title or abstract, and the medical subject heading “Seroepidemiologic Studies” be used when the report is of a population-based serological survey.	☑	Abstract
Introduction	2	ROSES-I 2.1: State what is known about the kinetics of antibody rise, decay, and persistence following infection for the particular virus being studied and the justification for threshold antibody titers or changes in titers used to define evidence of infection.	☐	Not provided
		ROSES-I 2.2: State what is known about the sensitivity and specificity of the antibody detection assay being used.	☐	Not provided
	3	ROSES-I 3.1: State the specific measure of occurrence that is being estimated, for example, point seroprevalence, cumulative incidence of infection, secondary infection risk.	☑	Somewhat though in case of vaccination not antibody titre.
**Epidemiologic methods**				
Study design	4	ROSES-I 4.1: State which specific seroepidemiologic study design was chosen and why.	☑	Has been mentioned in Section 2.2.

Setting	5	ROSES-I 5.1: Describe the timing of the biological sampling in relation to the disease epidemiology in the study population (the beginning, peak, and end of virus transmission).	☐	Not applicable.
		ROSES-I 5.2: Where known, describe the timing of biological sampling in individuals in relation to disease onset and to exposures of interest.	☐	Not applicable
		ROSES-I 5.3: State the interval between sequential biological samples (serial cross-sectional or longitudinal studies), or specify whether only a single sample was collected (crossectional study).	☑	Only one sample was collected. Has been mentioned in Section 2.2.
Participants	6	ROSES-I 6.1: For case-ascertained transmission studies, describe the method of case ascertainment and criteria for defining a "case".	☐	Not applicable
		ROSES-I 6.2: For household-or institution-based transmission studies, describe the definition of a household or the institution.	☐	Not applicable
		ROSES-I 6.3: For outbreak investigations involving serologic sampling, describe the setting in which the cases were identified, for example, village/ residential setting, occupational workplace.	☐	Not applicable. Vaccination study was carried out within the occupational workplace, i.e. Jahangirnagar University
		ROSES-I 6.4: To aid the interpretation of seroepidemiologic studies of novel influenza A virus subtypes, the results from exposed populations should be compared with the results from unexposed populations. Efforts to validate the assay in virologically confirmed cases should be reported.	☐	Not applicable

Variables	7	ROSES-I 7.1 The median age and range for each exposure group should be reported.	☑	Section: Table-1
		ROSES-I 7.2: Describe the potential for immunization (specify vaccine and timing of vaccination in relationship to collection of serum), if applicable, to affect the outcome measures.	☑	Section: Table-1
		ROSES-I 7.3: Describe any known or potential immunological cross-reactivity that may bias the outcome measures.	☑	Section: Discussion
		ROSES-I 7.4: Describe illness definitions and methods for ascertaining the presence or absence of clinical illness in subjects.	☐	Not defined
Data sources/ measurement biases	8a	ROSES-I 8.1: If relevant, describe measures taken to identify and record immunization history.	☑	Section: 2.2
Bias	9	ROSES-I 9.1: If relevant, describe efforts to control for the potential effect of immunization on estimates of outcomes.	☐	Not provided.
Study size	10	ROSES-I 10.1: Describe the baseline estimated seroprevalence at given antibody titers or incidence of infection and cite published literature to support these estimates.	☑	Section: 2.2, 3.3
Quantitative variables	11	ROSES-I 11.1: Describe the serological assay's limit of detection and how this limit is defined or calculated. Describe how samples with a result below or on the borderline of the limit were handled in the analysis.	☑	Section: Discussion

		ROSES-I 11.2: Describe and justify the titer or other result used to define “seropositivity,” or the antibody titer change or change in other assay result used to define “seroconversion.” Avoid the term “seroconversion” unless referring to change from undetectable to detectable antibody level. Otherwise report the fold-rise in titer. Avoid the term “infection” but report “seroprevalence at a titer of ….”.	☑	Section: Results
		ROSES-I 11.3: If statements or inferences are made about protection from infection, describe what is known about the correlation between the assay results and protection from infection and illness.	☐	Not applicable. Our study do not comment on protection from infection but only look at the anitbody levels.
Statistical methods	12	ROSES-I 12.1: if relevant, state how the non-independence of data was managed.	☐	Not applicable.
		ROSES-I 12.2: if relevant, report methods used to account for the probability of seropositivity or seroconversion if infected, and to account for decay in antibody titers over time.	☑	Section: Materials and methods
**Laboratory methods**	**12a**			
Sample type and handling		ROSES-I 12a.1: Describe the sample type—serum or plasma. If plasma is used, specify the anticoagulant used (heparin, sodium citrate, EDTA, etc.).	☑	Section: 2.2
Serological assays		ROSES-I 12a.2: Describe the specimen storage conditions (4°C, −20 °C, −80 °C). If frozen prior to the analysis, describe the time to freezing and the number of freeze/thaw cycles prior to testing.	☑	Section: 2.2
		ROSES-I 12a.3: Specify the assay type (e.g., hemagglutination inhibition; virus neutralization/microneutralization; ELISA; other) and methods used to determine the endpoint titer.	☑	Section: 2.3

	ROSES-I 12a.4: Reference a previously published, CONSISE consensus serologic assay or WHO protocol if used, and any modifications of the protocol. If a previously published protocol is not used, provide full details in supplementary materials.	☐	Not applicable.
	ROSES-I 12a.5: State what is known about the determinants of the variability of the antibody detection assay being used.	☐	Not provided.
	ROSES-I 12a.6: Specify the antigen(s) used in the assay, including virus strain name, subtype, lineage or clade, with standardized nomenclature and reference; specify whether live virus or inactivated virus was used (where applicable).	☑	Section: 2.3
	ROSES-I 12a.7: Report if antigen(s) from potentially cross-reactive pathogens/strains were used in order to identify cross-reactivity, and specify which antigen was used, including virus name, subtype, strain, lineage and clade, with standardized nomenclature and reference.	☐	Not applicable.
	ROSES-I 12a.8: If red blood cells were used for a hemagglutinin inhibition assay, specify the animal species from which they were obtained and concentration (v/v) used.	☐	Not applicable.
	ROSES-I 12a.9: Describe positive and negative controls used.	☐	Section: 2.3
	ROSES-I 12a.10: Describe starting and end dilutions.	☐	Section: 2.3

		ROSES-I 12a.11: Specify laboratory biosafety conditions.	☐	Not provided. The ELISA test was carried out for determination of antibody titer.
		ROSES-I 12a.12: Specify whether replication was performed, and if so, the acceptable replication parameters.	☐	Not provided. However, for each ELISA sample a replication of 3 was carried out.
		ROSES-I 12a.13: Specify whether a confirmatory assay was performed and all specifics of this assay, at the same level of detail.	☐	Not applicable.
		ROSES-I 12a.14: Specify international standards used, if appropriate.	☐	Not provided.
**Results**				
Participants	13a	ROSES-I 13a.1: Report the numbers of individuals at each stage of the study—the numbers potentially eligible, examined for eligibility, confirmed eligible, included in the study, completing follow-up, and analyzed.	☑	Section: 3.1
		ROSES-I 13a.2: Give reasons for non-participation at each stage.	☐	Not provided.
		ROSES-I 13a.3: Consider use of a flow diagram	☐	Not provided.
Descriptive data	14a	ROSES-I 14a.1: Give characteristics of study participants (e.g., demographic, clinical, social) and information on exposures and potential risk factors.	☑	Section: 3.1
		ROSES-I 14a.2: Indicate the number of participants with missing data for each variable of interest.	☑	Section 3.1

		ROSES-I 14a.3: Cohort study—summarize follow-up time (e.g., average and total amount).	☐	Not applicable.
Outcome data	15a	ROSES-I 15a.1:Cohort study—report the numbers of outcome events or summary measures over time<br>Case–control study—report the numbers in each exposure category, or summary measures of exposure<br>Cross-sectional study—report the numbers of outcome events or summary measures.	☑	Section: 3
Main results	16	ROSES-I 16.1: Report unadjusted estimates of distribution of titers by age group.	☑	Section: 3.9
		ROSES-I 16.2: Report methods to standardize the results from the study sample to the target population.	☑	Section: 3
Other analyses	17	ROSES-I 17.1: Report other analyses performed—analyses of subgroups and interactions, and sensitivity analyses.	☑	Section: 3.3, 3.4, 3.5, 3.6, 3.7
**Discussion**				
Key results	18	ROSES-I 18.1: Summarize key results with reference to study objectives.	☑	
Limitations	19	ROSES-I 19.1 Discuss limitations and strengths of the study with reference to Table 1.	☑	
Interpretation	20	ROSES-I 20.1: Discuss the interpretation of the results in the context of known or potential cross-reactivity.	☐	Not provided.

Generalizability	21	ROSES-I 21.1: Discuss the generalizability (external validity) of the study results.	☑	
**Other information**				
Funding	22	ROSES-I 22.1: Specify if institutional review board approval was received; if not, specify reason (e.g., public health outbreak response/non-research designation).	☑	No fudning was used for the research.
